# Determination of Optimal Magill Forceps Hand Position and Laryngoscope Type to Remove a Simulated Foreign Body Airway Obstruction

**DOI:** 10.5811/westjem.49101

**Published:** 2026-05-18

**Authors:** Michael Berkenbush, Coco Thomas, Michael Mysh, John Rutledge, Raymond Dwyer, Scott Kansky

**Affiliations:** * Morristown Medical Center, Department of Emergency Medicine, Morristown, New Jersey; † University of Pittsburgh, Department of Emergency Medicine, Pittsburgh, Pennsylvania; ‡ Hackensack Meridian School of Medicine, Nutley, New Jersey; § Atlantic Center for Research, Morristown, New Jersey; || Atlantic Mobile Health, Florham Park, New Jersey

## Abstract

**Introduction:**

Foreign body removal for airway obstruction is an infrequent skill performed by paramedics, and Magill forceps for removal of a foreign body from the airway is reserved for patients with persistent obstruction. Traditional paramedic instruction uses direct laryngoscopy to visualize foreign body removal. Our objective in this study was to evaluate time to removal of an obstruction comparing hand position and hyperangulated video laryngoscopy vs direct laryngoscopy with Magill forceps.

**Methods:**

A foreign body airway obstruction station was included in paramedics’ annual competency assessment over a two-year period as a quality improvement project. Paramedics were randomized to remove the foreign body with either direct laryngoscopy or hyperangulated video laryngoscopy with a handheld video laryngoscope. Our primary outcome measure was time from blade insertion to foreign body removal. The paramedics had further training regarding hand position prior to the second year’s annual competency assessment. After fitting a mixed-effects statistical model to the data, we included fixed effects of competency assessment year, device used, hand position, and random effect of paramedic. We evaluated the significance of these predictors on the dependent variable of time for removal.

**Results:**

We observed 245 foreign body airway obstruction removals, 77 in year 1 and 168 in year 2. The direct laryngoscopy (n = 123) and hyperangulated video laryngoscopy (n = 122) groups were nearly equal. During year 1, hand position was noted to be an important factor for removal time, as paramedics were seen to use different hand positions. Recording of hand position began during year 1, which excluded 95 earlier observations from year 1. Competency training year was not a significant factor for time to removal. However, direct laryngoscopy was faster at 14.9 seconds [sec], (95% CI 12.9–17.1) than hyperangulated video laryngoscopy at 19.2 sec [16.9–22]; *P* < .001. Of the four possible hand positions, forceps grasped by the right thumb and either middle or fourth finger, with the hand in a handshake position, and with the forceps superior to the hand was associated with the shortest time to foreign body removal. This optimal hand position was significantly better than the other grasping methods, with a mean of 11.2 sec (95% CI, 10.2–12.4) vs underhand method, 17.5 sec (14.7–21); overhand method, 15.1 sec (10.7–21.2); and multiple positions (27.5 sec, 22–34.5); *P* < .001).

**Conclusion:**

We found that hand positioning for Magill forceps significantly affected time to foreign body removal. In addition, direct laryngoscopy outperformed hyperangulated video laryngoscopy. Given these findings, training on removal of foreign body airway obstruction with Magill forceps should emphasize optimal hand positioning. Paramedics may also consider the use of direct laryngoscopy over hyperangulated video laryngoscopy in removal of these obstructions. Further research is required to validate these findings.

## INTRODUCTION

Foreign body removal for airway obstruction is an infrequent skill performed by paramedics. At our Advanced Life Support (ALS) agency, over the past five years there has been an average of four cases per year of foreign body airway obstruction requiring management by our paramedics, of about 14,000 ALS patient contacts per year. In cases of foreign body removal for airway obstruction, the use of Magill forceps is reserved for those with persistent obstruction, despite adequate first-line interventions. When Basic Life Support fails, ALS management includes removal of the visualized foreign body by laryngoscopy, advanced airway management, and cardiopulmonary resuscitation if indicated.^1^ Traditional paramedic instruction uses direct laryngoscopy to facilitate foreign body removal.

Magill forceps were initially developed to facilitate nasotracheal intubation.^2^ They are constructed with “a bend to clear the field of vision… the ends which grasp the catheter representing a cylinder split longitudinally and serrated on the inner surface.”^3^ Today, Magill uses have expanded to include removal of foreign body for airway obstruction in the unconscious patient by paramedics. Use of Magill forceps has been shown to be associated with favorable outcomes in foreign body obstruction refractory to the Heimlich maneuver and in out-of-hospital cardiac arrest due to presence of a foreign body.^4^–^6^ Our objectives in this study were to 1) compare the efficacy of direct laryngoscopy vs hyperangulated video laryngoscopy when using Magill forceps for foreign body removal for airway obstruction, and 2) assess the effect of hand position on time to removal with Magill forceps.

## METHODS

### Study Setting and Design

We conducted this study at a large, hospital-based emergency medical services (EMS) agency with over 150 paramedics in northern New Jersey. A foreign body airway obstruction station was included in annual internal competency training over a two-year period. All paramedics were assessed in technique for removal of a foreign body using simulation with a manikin in a skills lab. Participants were randomized into two cohorts for method of removal with Magill forceps: direct laryngoscopy and hyperangulated video laryngoscopy. Participants were given a set of Magill forceps and a direct laryngoscope Miller or Macintosh blade of their choice (direct laryngoscopy group) or a GlideScope Go device (Verathon Inc, Bothell, WA) (hyperangulated video laryngoscopy group) for retrieval.

For retrieval simulation, a cork was shaped to fit into a manikin’s tracheal opening above the vocal cords to simulate an airway foreign body. The cork was placed under direct visualization by researchers with good reproducibility. In rare instances where the cork was malpositioned into the trachea during the exercise, the manikin was reset and the retrieval was repeated. Year 1 data was collected during an annual skills competency assessment. Approximately 18 months later, year 2 data was collected at another annual skills competency assessment. Between year 1 and year 2 data collection, an educational intervention was delivered to all agency paramedics, with instruction focusing on technique when using Magill forceps. This quality improvement study was deemed exempt per the institutional review board, as it was developed with the aim of local improvement at our agency, based on system policy SOP-HRP-031 that states “a project does not meet the definition of human subject research if it is limited to program evaluation, quality improvement or quality assurance activities designed specifically to assess or improve performance within the department.”

**Population Health Research Capsule**

What do we already know about this issue?

*Current guidelines and paramedic instruction include foreign body airway obstruction removal with Magill forceps*.

What was the research question?

*We compared the efficacy of laryngoscope type and different hand positions when using Magill forceps for removal of foreign body airway obstruction*.

What was the major finding of the study?

*Direct laryngoscopy removal at 14.9 seconds (12.9–17.1) was faster than hyperangulated video laryngoscopy at 19.2 sec (16.9–22), P < .001. The “handshake” position was optimal*.

How does this improve population health?

*Foreign body airway obstruction is a time-sensitive condition. Proper techniques in using the Magill forceps can ensure timely foreign body removal*.

### Study Population

All agency paramedics were included in this study during annual training and were the majority of the participants. Other ALS clinicians such as critical care transport nurses (who also may work on 9-1-1 paramedic units) and flight team clinicians were also included in the training. A total of 172 paramedics were assessed in year 1 and 168 paramedics in year 2.

### Data Collection and Analysis

Data collected included time for removal, visualization technique, hand position on Magill forceps, and documentation of whether there were multiple hand-position changes. Observation and interim analysis during year 1 suggested that hand positioning was an important variable; therefore, recording of this variable began approximately halfway through that competency session. Paramedics were randomized between the direct laryngoscopy and hyperangulated video laryngoscopy groups using a spreadsheet randomizer. The competency facilitator measured the time from blade insertion to foreign body removal using a smartphone stopwatch. Data was abstracted into an Excel spreadsheet (Microsoft Corporation, Redmond, WA) for analysis. A mixed-effects statistical model was fit to the data. Included in the statistical model were fixed effects (competency assessment year, device used, and hand position) and random effects (paramedic). The significance of these predictors on the dependent variable of time for removal was subsequently evaluated. *P* values < .05 were considered statistically significant. We used IBM SPSS Statistics v29 (International Business Machines Corporation, Armonk, NY) for all data analyses.

### Educational Intervention

**Figure 1:**
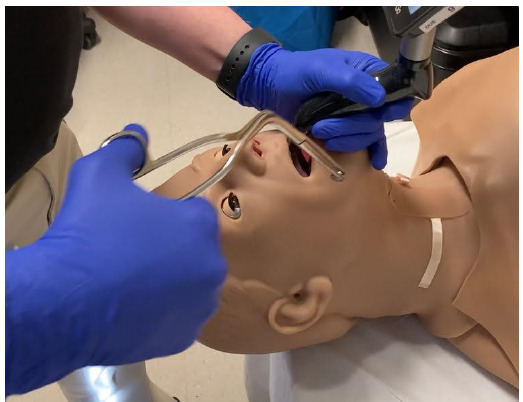
Optimal positioning of the Magill forceps in a study comparing hand position and use of hyperangulated video laryngoscopy vs direct laryngoscopy for foreign body removal for airway obstruction.

Paramedics were not trained on hand position for removal of foreign body for airway obstruction prior to data collection in year 1. Results from the initial analysis after year 1 demonstrated that different hand positioning produced varied results for time from blade insertion to foreign body removal, with a superior technique identified (see Figure 1). Therefore, an educational intervention was developed that included a brief presentation discussing optimal hand positioning along with a hands-on skills station to reinforce the optimal technique. This intervention was delivered to paramedics at a skills session about 12 months prior to the year 2 annual competency training.

## RESULTS

There were 245 valid observations over the two years of competency sessions. We excluded 95 paramedics from year 1 as hand position had not been recorded. The number of paramedics included in each laryngoscopy method group was nearly equal (direct laryngoscopy = 123; hyperangulated video laryngoscopy = 122). Four groupings of hand positions were noted during data collection: the optimal “handshake” position; overhand; underhand; and multiple positions (as further described in Appendix 1). See Table 1 for the dataset description.

The removal time data was not normally distributed; therefore, we performed a log transformation prior to analysis. Competency assessment year was not a significant predictor of time to complete the procedure (*P* = .53). Laryngoscopy device used and Magill forceps hand position were both significant predictors of time to complete the procedure. See Table 2 for the results.

**Figure 2:**
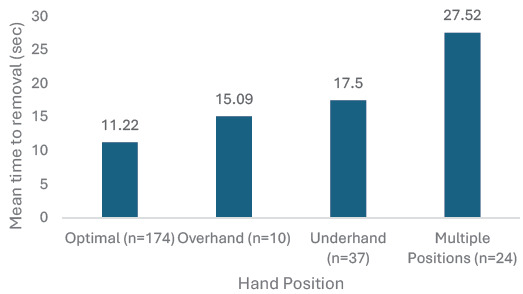
Mean time to foreign body removal based on hand position of Magill forceps in a study comparing use of hyperangulated video laryngoscopy vs. direct laryngoscopy for foreign body removal for airway obstruction.

The laryngoscopy device used was a significant factor in time to removal, with direct laryngoscopy removal at a mean of 14.9 seconds (95% CI, 12.9–17.1) compared to hyperangulated video laryngoscopy at 19.2 seconds (16.9–22), *P* < .001. The optimal “handshake” position significantly outperformed the other described methods, with a mean of 11.2 seconds (10.2–12.4) compared to the underhand position (17.5 seconds, 14.7–21), overhand position (15.1 seconds, 10.7–21.2), and multiple positions (27.5 seconds, 22–34.5), *P* < .001 (Figure 2).

**Figure 3:**
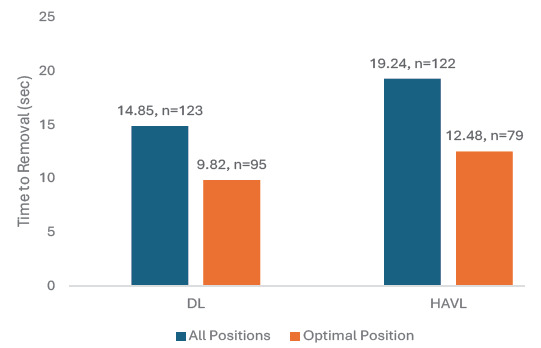
Mean time to removal of foreign body for airway obstruction using direct laryngoscopy vs hyperangulated video laryngoscopy, with all hand positions compared to the optimal position. *DL*, direct laryngoscopy; *HAVL*, hyperangulated video laryngoscopy; *sec*, second.

The mean time for removal was calculated for the optimal hand position based on laryngoscopy technique, including data from year 1 and year 2. For direct laryngoscopy, the mean time for removal with the optimal hand position was 9.82 sec (95% Cl 8.7–11.08). For hyperangulated video laryngoscopy, the mean time for removal was 12.48 seconds (11.16–13.95). Figure 3 shows direct laryngoscopy vs hyperangulated video laryngoscopy for all positions and the optimal position.

## DISCUSSION

Information is limited on the correct technique and use of Magill forceps for removal of a foreign body obstructing the airway, and there is not a widely accepted standard or protocol for their use in EMS. Review of current literature showed reference documents that include indications for Magill forceps use in removal of foreign body from the airway, patient positioning, laryngoscopy technique, and removal technique.^6^, ^7^ We found a single EMS protocol that has images of how to hold Magill forceps and demonstrates technique.^8^ One text notes the appropriate hand position and grip while using the forceps, although the technique described was used for nasotracheal intubation, rather than for removal of a foreign body from an obstructed airway.^9^ No studies were found that demonstrated specifically how to hold the Magill forceps for removal of foreign body from the airway.

There are scholarly papers that demonstrate the utility of Magill forceps for the removal of a tracheal foreign body. One study, based in Osaka, Japan, found that the use of Magill forceps for out-of-hospital cardiac arrest due to foreign body obstruction was associated with neurologically favorable outcomes.^4^ Another study demonstrated that use of Magill forceps was successful in cases of foreign body aspiration refractory to the Heimlich maneuver.^5^ Additionally, there is a case report where Magill forceps were used for near-complete foreign body airway obstruction with respiratory distress.^6^ None of these articles describe the technique for holding and positioning the forceps.

One prior study compares the use of the Macintosh laryngoscope and the GlideScope device for removal of a hypopharyngeal foreign body.^10^ In that study, a lightly embalmed cadaver was used for simulation. The Macintosh blade (direct laryngoscopy) demonstrated superior efficiency for foreign body removal when compared to the GlideScope (hyperangulated video laryngoscopy). However, the study does not address the hand positioning of the paramedic attempting the extraction with Magill forceps. The GlideScope device has been demonstrated to be superior to DL for first pass success in endotracheal intubation.^11^ By policy, our paramedics use hyperangulated video laryngoscopy for all intubation attempts. Despite paramedic familiarity with hyperangulated video laryngoscopy, this did not lead to faster foreign body removal times with video assistance, compared to direct laryngoscopy. One consideration is that while video laryngoscopy is ideal for visualizing deeper airway structures, direct laryngoscopy may be better for visualizing the hypopharynx.

The importance of optimal hand positioning when using Magill forceps removal of foreign body from the airway is clear. Although Magill forceps have found their way into the toolbox of most EMS agencies, there has not been prior investigation into the most effective hand position for this infrequently used tool. The low frequency of cases that require extraction with the Magill forceps also makes it difficult to study in a prospective manner. The description of the optimal use and hand positioning as described can help to educate paramedics and guide the development of protocols for Magill forceps use in EMS.

## LIMITATIONS

There are several limitations to this study. Because removal of a foreign body for airway obstruction is a rare procedure in the field, a training model was used. The use of manikins for this study limits external validity, given the variable and diverse presentations of foreign bodies causing airway obstruction that are seen in the field. The model used was reproducible to allow for accurate comparison; however, the composition of a true foreign body can be highly variable, and the use of a cork may not approximate real foreign bodies that are slippery and more difficult to grasp. To ensure standardization, the manikin’s head was placed in a neutral position prior to beginning the procedure. It is possible that neutral positioning could affect airway axis alignment and the ease of using direct laryngoscopy, although the paramedic could manually reposition the head to improve alignment.

In addition, removal of a foreign body for airway obstruction is typically done on the floor, while this training was done with the manikin positioned on a table. Measurement bias may have been introduced by using an unblinded timing device. Additionally, hand positioning data was incomplete from year 1, as data was not collected for the entire cohort, prior to identifying its importance. Also, it is possible that a small number of paramedics did not receive the educational intervention due to employee turnover. Although this study does not include patient-oriented outcomes, it can serve as a step toward establishing a standardized technique for the use of Magill forceps.

## CONCLUSION

We found that direct laryngoscopy resulted in faster removal of a foreign body from the airway in comparison to hyperangulated video laryngoscopy. Paramedic hand position also had a significant impact on time to removal. Along with improving familiarity with the Magill forceps, it is important to train paramedics on a standard method for grasping the Magill forceps, as our research supports a superior technique (Figure 1). Further research is required to validate these findings.

## Supplementary Information



## Figures and Tables

**Table 1:** Paramedic observation data obtained during annual competency session in a study comparing hand position of Magill forceps and hyperangulated video laryngoscopy vs. direct laryngoscopy for foreign body removal for airway obstruction.

		Label	Count (n)
Competency year	1	Year 1	77
	2	Year 2	168
Laryngoscopy device used	Direct laryngoscopy		123
	HAVL		122
Magill forceps hand position	1	Optimal	174
	2	Underhand	37
	3	Overhand	10
	4	Multiple positions	24
Valid observations			245
Excluded (no hand-position data)			95
Total			340

*HAVL*, hyperangulated video laryngoscopy.

**Table 2:** Mean time for foreign body removal for airway obstruction based on fixed effects: year, device, and hand position.

		Mean time (sec)	95% CI for the mean	P value
Competency assessment year	Year 1	17.32	15.13 – 19.83	0.53
	Year 2	16.49	14.30 – 19.03	
Laryngoscopy device used	Direct laryngoscopy	14.85	12.91 – 17.06	< .001
	HAVL	19.24	16.86 – 21.96	
Magill forceps hand p-osition	Optimal	11.22	10.18 – 12.38	< .001
	Underhand	17.50	14.66 – 20.91	
	Overhand	15.09	10.71 – 21.26	
	Multiple positions	27.52	21.98 – 34.47	

*HAVL*, hyperangulated video laryngoscopy.
