# Supplementary material for: Determination of Optimal Magill Forceps Hand Position and Laryngoscope Type to Remove a Simulated Foreign Body Airway Obstruction
Source: West J Emerg Med. 2026 May 18;27(3):709–14. doi: 10.5811/westjem.49101 (PMC13246202; doi:10.5811/westjem.49101)
Supplement: Supplementary file 1 [file wjem-27-709-s001.docx]

Appendix 1

***1. Optimal Method***

The first position used is denoted as the “optimal” method. The forceps are grasped with the right thumb and middle finger or fourth finger, with the right hand in a handshake position, and the angle of the forceps superior to the hand. This technique is demonstrated in Figure 1. This positioning allows for horizontal orientation of the grasping end at the glottic opening, therefore minimizing the risk of damage to the epiglottis during removal. The field of vision is clear with the hand below the angle of the forceps. Additionally, the hand can be easily rotated 180 degrees, so that the grasping end can be oriented vertically if needed, without interference from the laryngoscope. Paramedics who used this hand position demonstrated the fastest foreign body removal times.

***2. Overhand Method***

A common pre-conceived notion is that the angle of the forceps should be aligned with the natural curvature of the airway. However, this is incorrect, as the bend in the Magill forceps does not actually enter the airway. With this positioning, the angle does not assist with visualization or the extraction of the foreign body. Figure 4 provides a visualization of the “overhand method,” where the hand is superior to the angle of the forceps during extraction. Here, the forceps are again grasped by the right thumb and middle finger or fourth finger, with the right hand in a handshake position, but the angle of the forceps is inferior to the hand.

This positioning causes the grasping end of the forceps to be positioned vertically within the airway. For foreign bodies in the hypopharynx, this method may yield success. However, if the foreign body is located near the glottic opening, there is a higher risk of epiglottis trauma during a removal attempt. Additionally, the range of rotation is limited to 90 degrees in this position, offering poor maneuverability. Rotating the forceps in a counterclockwise manner causes interference with the laryngoscope.


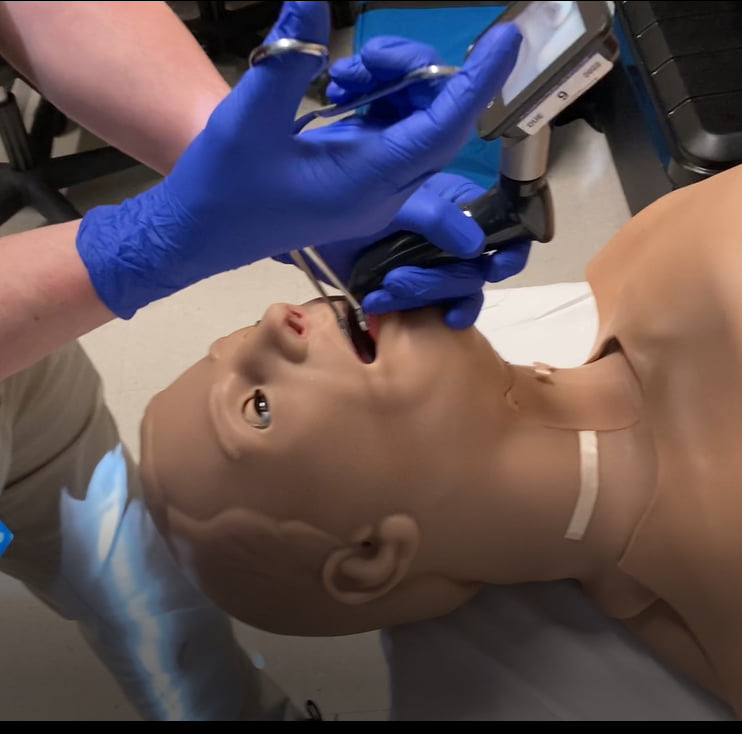


Figure 4: Demonstration of the overhand method

***3. Underhand Method***

The final method noted also has the angle of the forceps inferior to the hand. In this case, the hand was in varied positions: supine, pronated, or flexed. Each of these three positions used a similar backhanded wrist motion to insert the forceps, therefore they were grouped together and denoted as the “underhand method.” Figure 3 shows an example of an underhand method.

In each of the underhand positions, the grasping end of the forceps are oriented horizontally relative to the glottic opening. The ability to rotate the forceps is impaired by the laryngoscope and rotation limits the field of vision, thus making this an inferior method for foreign body removal.


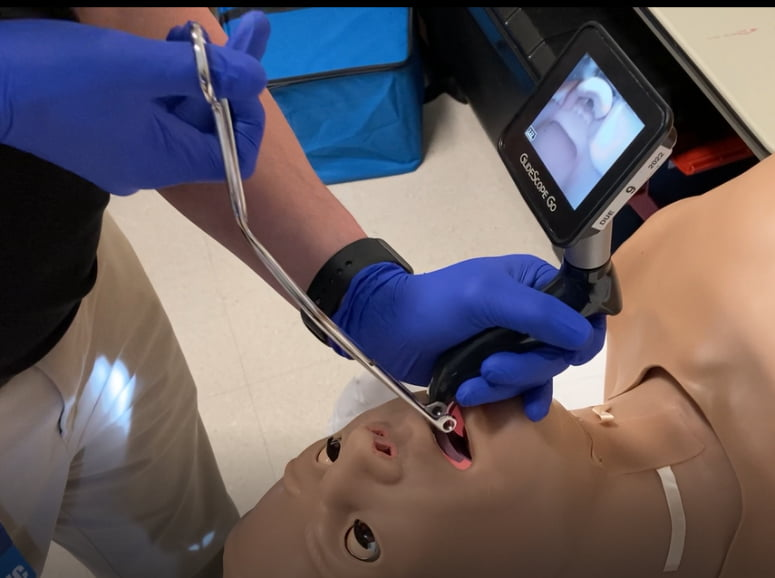


Figure 5: One example of the underhand method

***4. Multiple Positions***

The last group of hand positions in our cohort were paramedics who used multiple hand positions. This group started using any of the above techniques but switched hand positions during the removal. Many paramedics cited inexperience with Magill forceps and long length of time since they last used the forceps when reflecting on their hand positioning. Paramedics who used multiple techniques had the longest removal times compared to the other groups.
